# Supplementary material for: A comparison of chain-of-thought reasoning strategies across datasets and models
Source: PeerJ Comput Sci. 2024 Apr 30;10:e1999. doi: 10.7717/peerj-cs.1999 (PMC11157560; doi:10.7717/peerj-cs.1999)
Supplement: Supplemental Information 1 — The paper employs Krippendorff’s Alpha as the primary performance metric. In these supplementary tables, all performance values are presented in terms of accuracy for the sake of completeness. [file peerj-cs-10-1999-s001.pdf]

S1 SUPPLEMENTARY TABLES

Prompt	Model avg. accuracy (CI) n per prompt = 1188
Zhou	**.68** (.65, .70)
Articulate	.67 (.64, .70)
Rephrase	.67 (.64, .69)
Elaborate	.66 (.63, .69)
Zhou-instruction	.65 (.63, .68)
Plan	.65 (.62, .68)
Kojima	.64 (.62, .67)
Direct	.64 (.61, .67)
Self-critique	.64 (.61, .67)
Converse	.64 (.61, .66)

Table S1. Accuracy of prompts. Accuracy of prompts averaged over datasets. In Table 3, text corresponding to the prompt names can be found. Average taken over all six models. N total = 11880.

Dataset	Accuracy (CI) n per dataset = 1980	Base Rate
WorldTree v2	**.88** (.86, .89)	.25
CommonsenseQA	.77 (.75, .79)	.2
OpenBookQA	.74 (.72, .76)	.25
StrategyQA	.67 (.65, .69)	.5
MedMCQA	.49 (.46, .51)	.25
MedQA	.38 (.36, .40)	.2

Table S2. Accuracy on datasets. Accuracy on datasets averaged over models and prompts. Base rate for random chance, dependent on number of answer choices in datasets. N total = 11880

Model	Accuracy (CI) n per model = 1980
GPT-4	**.85** (.83, .86)
GPT-3.5-turbo	.74 (.72, .76)
Davinci-003	.63 (.61, .65)
Flan-T5-XXL	.61 (.59, .63)
Davinci-002	.59 (.56, .61)
Command-XL	.52 (.50, .55)

Table S3. Accuracy of models. Accuracy of models averaged over datasets and prompts. N total = 11880.

dataset<br>prompt	CommonsenseQA	MedQA	MedMCQA	OpenBookQA	StrategyQA	WorldTree v2
Direct	.74 (.68, .81)	.38 (.31, .45)	.46 (.39, .53)	.74 (.68, .80)	.64 (.57, .71)	.88 (.84, .93)
Kojima	.75 (.69, .81)	.39 (.32, .46)	.44 (.37, .51)	.71 (.65, .78)	**.73** (.67, .80)	.85 (.79, .90)
Zhou	.78 (.72, .84)	.40 (.33, .47)	**.54** (.47, .61)	**.81** (.75, .87)	.67 (.60, .74)	.87 (.83, .92)
Plan	.78 (.73, .84)	.36 (.30, .43)	.48 (.41, .56)	.74 (.68, .80)	.66 (.59, .73)	.87 (.82, .91)
Articulate	.78 (.72, .84)	.39 (.32, .46)	.52 (.44, .59)	.76 (.70, .82)	.66 (.59, .73)	**.91** (.87, .95)
Rephrase	**.80** (.75, .86)	.38 (.31, .45)	.49 (.42, .56)	.71 (.64, .77)	.72 (.65, .78)	**.91** (.87, .95)
Elaborate	.75 (.68, .81)	**.41** (.34, .48)	.53 (.46, .60)	.74 (.67, .80)	.68 (.61, .75)	.87 (.82, .92)
Converse	.71 (.64, .78)	.37 (.30, .44)	.51 (.43, .58)	.73 (.67, .79)	.66 (.59, .73)	.84 (.79, .89)
Self-critique	.79 (.73, .84)	.37 (.30, .43)	.44 (.37, .51)	.75 (.69, .81)	.62 (.55, .69)	.87 (.82, .92)
Zhou-instruction	.79 (.73, .85)	.37 (.30, .44)	.45 (.38, .52)	.74 (.68, .81)	.66 (.59, .73)	.90 (.86, .94)

Table S4. Accuracy of prompts per dataset. Accuracy of prompts per dataset averaged over models. Average over 198 items per promt/dataset pair. N total = 11880.

model<br>prompt	Command-XL	Flan-T5-XXL	GPT-3.5-turbo	GPT-4	Davinci-002	Davinci-003
Direct	.47 (.40, .54)	.62 (.55, .69)	.75 (.69, .81)	.81 (.76, .87)	.59 (.52, .66)	.61 (.54, .68)
Kojima	.45 (.38, .52)	.62 (.55, .69)	**.76** (.70, .82)	.86 (.81, .91)	.56 (.49, .63)	.61 (.54, .68)
Zhou	.55 (.48, .62)	.58 (.51, .65)	.75 (.69, .81)	**.89** (.84, .93)	**.65** (.58, .71)	**.66** (.59, .73)
Plan	.53 (.46, .60)	.62 (.55, .69)	.73 (.66, .79)	.84 (.79, .89)	.55 (.48, .62)	.63 (.56, .70)
Articulate	.53 (.46, .60)	**.65** (.58, .71)	.74 (.67, .80)	.86 (.81, .91)	.61 (.54, .69)	.65 (.58, .72)
Rephrase	**.58** (.51, .65)	.62 (.55, .69)	.73 (.66, .79)	.84 (.79, .89)	.61 (.54, .68)	.63 (.56, .70)
Elaborate	.55 (.48, .62)	.59 (.52, .66)	.73 (.66, .79)	.84 (.79, .89)	**.65** (.58, .72)	.60 (.53, .67)
Converse	.52 (.45, .59)	.61 (.55, .68)	.71 (.65, .78)	.81 (.76, .87)	.53 (.46, .61)	.62 (.55, .69)
Self-critique	.51 (.43, .58)	.58 (.51, .65)	.72 (.65, .78)	.84 (.79, .89)	.57 (.50, .64)	.64 (.57, .71)
Zhou-instruction	.56 (.49, .63)	.59 (.52, .66)	.75 (.69, .82)	.85 (.80, .90)	.54 (.47, .61)	.65 (.58, .71)

Table S5. Accuracy of prompts per model. Accuracy of prompts per model averaged over datasets. Average over 198 items per promt/model pair. N total = 11880.
